# Bone Mineral Density and Hemoglobin Levels: Opposite Associations in Younger and Older Women

**DOI:** 10.3390/ijerph18105495

**Published:** 2021-05-20

**Authors:** Tzyy-Ling Chuang, Malcolm Koo, Mei-Hua Chuang, Yuh-Feng Wang

**Affiliations:** 1 Department of Nuclear Medicine, Dalin Tzu Chi Hospital, Buddhist Tzu Chi Medical Foundation, Chiayi 622401, Taiwan; b8601139@tmu.edu.tw; 2 School of Medicine, Tzu Chi University, Hualien 970374, Taiwan; 3 Graduate Institute of Long-term Care, Tzu Chi University of Science and Technology, Hualien City, Hualien 973302, Taiwan; m.koo@utoronto.ca; 4 Faculty of Pharmacy, National Yang-Ming University, Taipei 112304, Taiwan; cmh618@ms32.hinet.net; 5 Department of Pharmacology, School of Medicine, Tzu Chi University, Hualien 970374, Taiwan; 6 Center of Preventive Medicine, Dalin Tzu Chi Hospital, Buddhist Tzu Chi Medical Foundation, Chiayi 622401, Taiwan

**Keywords:** bone density, hemoglobin, hematologic diseases, female, cross-sectional studies

## Abstract

This cross-sectional, retrospective medical record review study aimed to investigate the association between hemoglobin levels and bone mineral density (BMD) in adult women. Medical records obtained from general health examinations conducted from June 2014 to July 2020 at a regional hospital in southern Taiwan were reviewed. Anthropometric and laboratory data were recorded. BMD of the lumbar spine and bilateral femoral neck regions was assessed by dual energy X-ray absorptiometry. Linear regression analysis was used to assess the association between BMD and hemoglobin level with and without adjusting for other anthropometric and laboratory data. The study included 9606 female patients with a mean age of 55.9 years. Of these, 2756 (28.7%) were aged ≤ 50 years and 6850 (71.3%) were aged > 50 years. Results from multiple linear regression analysis showed that hemoglobin and femoral and lumbar spine BMD were significantly correlated. A higher hemoglobin level was significantly associated with a lower BMD level in females aged ≤ 50 years, but with a higher BMD level in those aged > 50 years. Given the relationship between bone metabolism and hematopoiesis, additional research is needed to elucidate the association between hemoglobin and BMD levels in different age groups, particularly in premenopausal and perimenopausal women.

## 1. Introduction

Bone mineral density (BMD) value, acquired with dual-energy X-ray absorptiometry (DXA), is an estimation of the quantity of bone mass [1]. Women can experience pronounced bone loss during perimenopause and after menopause [2]. Low BMD and osteoporosis are major public health concerns, particularly among women, because of their association with an increased risk of bone fracture and subsequent morbidity, mortality, disability, and decreased quality of life, as well as social costs [3,4].

Anemia is defined as a reduction in the oxygen carrying capacity of blood due to decreases in hemoglobin level and red blood cell volume. Worldwide in 2011, an estimated 496 million nonpregnant and 32 million pregnant women had anemia [5]. A low concentration of hemoglobin is a public health concern that affects low-, middle-, and high-income countries. Anemia and low hemoglobin have been shown to be strong risk factors for increased mortality and disability [6,7], loss of physical performance [8], and poor quality of life [9]. They are also associated with a number of chronic diseases, including cardiovascular disease [10].

Many diseases and conditions can lead to low hemoglobin; these include malnutrition, chronic disease, malignant tumors, or chronic kidney disease [11]. In addition, the concurrence of osteoporosis and anemia has been reported in a number of diseases, such as renal failure [12], thalassemia [13], sickle-cell anemia [14], and chronic inflammatory conditions [15]. Hypoxemia has also been shown to be a risk factor for osteoporosis [16,17]. Furthermore, several studies have reported that low hemoglobin levels were inversely and independently associated with BMD [18,19].

In our previous study based on a retrospective medical chart review of male patients, we found that low serum hemoglobin may be an important predictor of bone mineral loss and the risk of both hip fracture and major osteoporotic fracture [20]. Given the interconnectedness of bone metabolism and hematopoiesis, we sought to characterize the association of hemoglobin levels with BMD in women, and to determine whether the association was similar in younger and older women.

## 2. Materials and Methods

### 2.1. Patients and Study Variables

In this cross-sectional, retrospective medical record review study, female patients aged 20 to 90 years who had undergone a general health examination from June 2014 to July 2020 at Dalin Tzu Chi Hospital, Taiwan, were reviewed. The study protocol was approved by the institutional review board of the study hospital (IRB No. B11001010), which was fully aware of the study design and had waived the requirement for obtaining informed consent from the patients due to the use of anonymized medical records.

Medical history (hypertension, diabetes mellitus, hyperlipidemia, and kidney disease), anthropomorphic characteristics (age, height, weight, and body mass index (BMI)), and laboratory data (systolic blood pressure (SBP), albumin, alkaline phosphatase (ALP), estimated glomerular filtration rate (eGFR), calcium, fasting glucose, glutamic oxaloacetic transaminase (GOT), high-density lipoprotein cholesterol (HDL-C), low-density lipoprotein cholesterol (LDL-C), total cholesterol, triglycerides, hemoglobin, smoking history, and alcohol use) were recorded (Table 1 and Table 2). Smoking was defined by whether a patient was currently smoking or had ever smoked. Alcohol use was defined by whether a patient consumed three or more units of alcohol daily. The laboratory of the study hospital was accredited by the Taiwan Accreditation Foundation. All laboratory data were analyzed using the Beckman Coulter DxC 700 AU Clinical Chemistry System (Beckman Coulter K. K., Mishima, Japan) or Beckman Coulter Automated Chemistry Analyzer AU5800 (non-sterile) (Beckman Coulter K. K., Mishima, Japan), and Sysmex automatic XN hematology analyzer (Sysmex Corp., Kobe, Japan). Patients with cancer history were excluded.

### 2.2. Measurement of Bone Mineral Density

BMD was assessed by DXA, which is routine for all patients undergoing general health examination in the study hospital. The DXA was conducted using a Discovery Wi DXA system (Hologic Inc., Marlborough, MA, USA). DXA has the advantages of being less costly and producing less radiation exposure than qCT. Moreover, its ability to identify patients at risk of fracture has been demonstrated in many epidemiological studies [21]. Absolute BMD values were calculated for all patients. The measured areas included the lumbar spine and bilateral femoral neck regions. Patients whose BMD measured areas containing metal materials was excluded. The same densitometer was used for all patients to ensure valid comparisons.

### 2.3. Statistical Analysis

All statistical analyses were performed using PASW Statistics for Windows, Version 18.0 (SPSS Inc., Chicago, IL, USA). Results were expressed as mean and SD, or number (percentage), as appropriate. Differences in means or proportions in anthropometric and clinical characteristics between patients categorized by age (≤50 years and >50 years) were tested by *t*-test and Chi-square test, as appropriate. This cutoff value for age was based on the mean age at natural menopause, which is 50.2 years (SD 4.0 years) in Taiwanese women [22].

Simple linear regression analysis was conducted to evaluate the association between BMD (separately for the lumbar spine, left, and right femoral neck) and the demographic and clinical characteristics for all patients and by age. In addition, multiple linear regression analyses were conducted using the built-in stepwise variable selection procedure to identify the independent factors associated with BMD of the three bone sites. Potential confounding factors, including age, BMI, smoking, alcohol use, SBP, albumin, ALP, eGFR, calcium, glucose, GOT, HDL-C, LDL-C, total cholesterol, and triglycerides, were evaluated in the multiple linear regression analysis. To minimize the effects of disease treatment on disease status and physiological condition, laboratory data (e.g., SBP) was considered in the multiple regression models instead of the associated disease state (e.g., hypertension). The beta values for all continuous laboratory variables were expressed as per 10-unit increments.

## 3. Results

### 3.1. Patient Characteristics

Data from 10,117 women were identified and collected from the medical record database. Three of them were younger than 20 years old and 440 had cancer history. In addition, 68 women had metal materials at the BMD measured sites. These 511 women were excluded from the study.

A total of 9606 female patients with a mean age of 55.9 years were included in the study. Of these, 2756 were aged ≤ 50 years (28.7%) and 6850 were aged > 50 years (71.3%). A significantly higher proportion of the older patient group had hypertension, diabetes mellitus, hyperlipidemia, and kidney disease. Conversely, a significantly lower proportion of these patients were smokers and alcohol users. The younger patient group had significantly lower BMIs, SBPs, and levels of ALP, calcium, glucose, GOT, LDL-C, total cholesterol, triglycerides, and hemoglobin. On the other hand, the younger patient group had a significantly higher eGFR and levels of albumin and HDL-C (Table 1 and Table 2).

Table 2 also shows the BMD of the three bone sites. The mean BMDs of the lumbar spine, right, and left femoral neck in the younger patient group were 0.99 g/cm^2^ (standard deviation (SD) 0.13), 0.72 g/cm^2^ (SD 0.11), and 0.73 (SD 0.11) g/cm^2^, respectively. All were significantly higher than those of the older patient group, which were 0.84 g/cm^2^ (SD 0.14), 0.61 g/cm^2^ (SD 0.10), and 0.62 g/cm^2^ (0.10), respectively.

### 3.2. Simple Linear Regression Analysis of the Factors Associated with Bone Mineral Density

#### 3.2.1. Bone Mineral Density of the Lumbar Spine

Results from the simple linear regression analysis showed that hemoglobin, age, hypertension, hyperlipidemia, kidney disease, SBP, ALP, calcium, GOT, HDL-C, LDL-C, and total cholesterol were significantly and inversely associated with lumbar spine BMD. Conversely, height, weight, BMI, smoking, alcohol use, and eGFR were significantly and directly associated with lumbar spine BMD (Table 3).

In the younger patient group, hemoglobin, albumin, ALP, eGFR, calcium, and HDL-C were significantly and inversely associated with lumbar spine BMD. Conversely, height, weight, BMI, hypertension, diabetes mellitus, hyperlipidemia, alcohol use, SBP, glucose, and triglycerides were significantly and directly associated with lumbar spine BMD. In the older patient group, age, ALP, and HDL-C were significantly and inversely associated with the lumbar spine BMD. Conversely, hemoglobin, height, weight, BMI, hypertension, diabetes mellitus, smoking, alcohol use, glucose, and triglycerides were significantly and directly associated with lumbar spine BMD (Table 3).

#### 3.2.2. Bone Mineral Density of the Right and Left Femoral Neck

Results from simple linear regression analysis showed that hemoglobin, age, hypertension, hyperlipidemia, SBP, ALP, calcium, GOT, HDL-C, LDL-C, and total cholesterol were significantly and inversely associated with right and left femoral neck BMD. Conversely, height, weight, BMI, smoking, alcohol use, and eGFR were significantly and directly associated with right and left femoral neck BMD (Table 4 and Table 5).

In the younger patient group, hemoglobin, age, albumin, ALP, calcium, and HDL-C were significantly and inversely associated with right and left femoral neck BMD. Conversely, height, weight, BMI, hypertension, diabetes mellitus, alcohol use, SBP, glucose, and triglycerides were significantly and directly associated with right and left femoral neck BMD (Table 4 and Table 5). In the older patient group, age, SBP, ALP, and HDL-C were significantly and inversely associated with both right and left femoral neck BMD. Conversely, hemoglobin, height, weight, BMI, diabetes mellitus, smoking, alcohol use, eGFR, glucose, LDL-C, total cholesterol, and triglycerides were significantly and directly associated with both right and left femoral neck BMD. Furthermore, calcium was also significantly and directly associated with left femoral neck BMD (Table 4 and Table 5).

### 3.3. Multiple Linear Regression Analysis of Hemoglobin and Bone Mineral Density

#### 3.3.1. Bone Mineral Density of the Lumbar Spine

In multivariable analyses, a lower hemoglobin level was significantly and independently associated with a higher level of lumbar spine BMD, after adjustment for potential confounding factors, including age, BMI, alcohol use, SBP, albumin, ALP, eGFR, glucose, HDL-C, LDL-C, and triglycerides (Table 6). However, when the multiple linear regression analysis was stratified by age, the associations diverged. In the younger patient group, a lower hemoglobin level was significantly and independently associated with a greater lumbar spine BMD, after adjustment for age, BMI, alcohol use, ALP, eGFR, calcium, and glucose. Conversely, in the older patient group, a lower hemoglobin level was significantly and independently associated with a lower lumbar spine BMD, after adjustment for age, BMI, alcohol use, SBP, albumin, ALP, eGFR, calcium, glucose, HDL-C, LDL-C, and triglycerides (Table 6). The adjusted *R^2^* of the older female patients (adjusted *R^2^* = 0.214) was higher than the younger female patients (adjusted *R^2^* = 0.148).

#### 3.3.2. Bone Mineral Density of the Right Femoral Neck

In multivariable analyses, hemoglobin level was not associated with the right femoral neck BMD, after adjustment for age, BMI, alcohol use, albumin, ALP, and glucose (Table 7). However, when multiple linear regression analysis was stratified by age, a significant but divergent association between groups was observed, similar to that of the lumbar spine. In the younger patient group, a lower hemoglobin level was significantly and independently associated with a higher right femoral neck BMD, after adjustment for age, BMI, alcohol use, albumin, ALP, glucose, and LDL-C. Conversely, in the older patient group, a lower hemoglobin level was significantly and independently associated with a lower right femoral neck BMD, after adjustment for age, BMI, alcohol use, albumin, ALP, calcium, glucose, and GOT (Table 7). The adjusted *R^2^* of the older female patients (adjusted *R^2^* = 0.224) was higher than the younger female patients (adjusted *R^2^* = 0.172).

#### 3.3.3. Bone Mineral Density of the Left Femoral Neck

In multivariable analyses of the study population, hemoglobin levels showed no significant association with left femoral neck BMD, after adjustment for age, BMI, alcohol use, albumin, ALP, and glucose (Table 8). Similar to the results for the right femoral neck BMD, a significant but divergent association was observed by age stratification. In the younger patient group, a lower hemoglobin level was significantly and independently associated with a higher left femoral neck BMD, after adjustment for age, BMI, alcohol use, albumin, ALP, and glucose. Conversely, in the older patient group, a lower hemoglobin level was significantly and independently associated with a lower left femoral neck BMD, after adjustment for age, BMI, alcohol use, albumin, ALP, calcium, and glucose (Table 8). The adjusted *R^2^* of the older female patients (adjusted *R^2^* = 0.231) was higher than the younger female patients (adjusted *R^2^* = 0.172).

## 4. Discussion

In this cross-sectional study of health examination data of adult females, we found different associations of hemoglobin with BMD for younger and older women. Hemoglobin and BMD showed an inverse association in females aged 50 years or younger, but a direct association in those older than 50 years. This divergent relationship was observed in the association of hemoglobin with the BMD of the lumbar spine, right femoral neck, and left femoral neck. We stratified our patients into two groups based on the mean age at natural menopause in Taiwan to compare the associations between hemoglobin and BMD. Menstrual history may serve as a surrogate for the adequacy of hormonal functioning and be a marker for bone status in younger women. At the lumbar spine, having more lifetime menstrual cycles has been associated with increased BMD [23].

First, our findings in older women are consistent with the literature. A study of 371 postmenopausal Turkish women reported that hemoglobin values were significantly correlated with femur BMD and spine BMD measured by dual energy X-ray absorptiometry (DXA). The study also found that the presence of anemia was an independent predictor of low trabecular BMD [24]. Similarly, a study of 338 healthy postmenopausal Korean women showed a direct and significant association between blood cell counts and BMD [25]. In addition, anemia and low hemoglobin levels were inversely and independently associated with bone mass and density, as assessed by peripheral quantitative computerized tomography (qCT). A study of 530 older Italian community-dwelling women, aged 65 to 102 years, showed that a lower hemoglobin level was significantly associated with a lower BMD of the trabecular and cortical components of the bone [18]. Another study based on data from the fourth and fifth Korea National Health and Nutrition Examination Survey indicated that hemoglobin levels are significantly associated with femoral neck BMD in menopausal women [26]. However, conflicting results have been shown in other studies. A retrospective study of 673 postmenopausal Chinese women showed that BMD was inversely correlated with hemoglobin and red blood cell counts. Moreover, hemoglobin levels in those with osteoporosis were significantly higher than in those without osteoporosis, and a decrease in BMD was accompanied by an increase in hemoglobin [27]. Finally, a prospective longitudinal study of 5888 community-dwelling adults aged over 65 years found no significant correlation between a decrease in hemoglobin level and BMD [28].

Second, few studies have focused on the association of hemoglobin and BMD in younger women. Our results showed that hemoglobin was inversely associated with BMD in younger women. This finding was consistent with that reported in a large South Korean survey study. Hemoglobin levels were inversely associated with whole-body BMD and lumbar spine BMD in premenopausal women [26]. However, a retrospective study comprised of 1020 women aged 20 to 49 years who had undergone a routine health examination at a general hospital in Korea found that a higher hemoglobin level was significantly associated with a higher BMD, both before and after adjustment for potential confounding factors. The discrepancy between this result and ours might be attributable to their exclusion of women diagnosed with and being treated for anemia [29]. In addition, a study based on secondary data analysis of the 2009–2010 Korea National Health and Nutrition Examination Survey showed no significant association between hemoglobin and BMD among 614 females aged 10 to 18 years, with or without adjustment for other potential confounders, in which BMD was measured at both the femur and lumbar spine using DXA [30]. In the present study, female patients younger than 20 years were excluded because peak bone mass is usually achieved in the late teenage years or early 20s. Future research should focus on exploring the association between hemoglobin and BMD in younger women.

As to the possible mechanisms linking hemoglobin and BMD, Fujimoto et al. proposed that increased oxidative stress and extracellular acidification under hypoxemia could interfere with bone remodeling and decrease BMD [16]. This hypothesis is in line with the observation of lower BMD levels among patients with thalassemia, in which the hematopoietic function is continuously hindered [31]. However, in the case of premenopausal women, iron-deficiency anemia as a result of periodic blood loss may promote bone formation.

Our study has some limitations. First, the causal relationship between hemoglobin levels and BMD could not be established with the cross-sectional design of this study. Second, because the data were collected from records of health examinations, not all associated variables of hemoglobin and BMD were available for analysis, such as medication use, age of menopause, ferritin, thyroid or parathyroid hormone, and vitamin D levels. Third, details of disease diagnosis were not available from the records of health examinations, such as information on the types of diabetes or hypertension in our patients. Fourth, the age of onset of menopause may be affected by certain chronic diseases, such as diabetes [32]. In the absence of patients’ estradiol levels, we categorized our patients operationally into a younger and older group based on the mean age of menopause in Taiwan.

## 5. Conclusions

Findings from our study showed that hemoglobin level was significantly correlated with femoral and lumbar spine BMD. A higher hemoglobin level was associated with a lower BMD level in females aged 50 years or younger, but with a higher BMD level in those older than 50 years. Given the relationship between bone metabolism and hematopoiesis, more work is needed to elucidate the association between hemoglobin and BMD levels across different age groups, as well as to evaluate the usefulness of hemoglobin measurement as an index for osteoporosis screening.

## Figures and Tables

**Table 1:** Demographic and clinical characteristics of the patients (N = 9606).

Variable	All Patients (N = 9606)	Age Group		*p*
		≤50 Years (n = 2756)	>50 Years (n = 6850)	
Age (years)	55.9 (11.0)	42.3 (6.9)	61.3 (7.0)	<0.001
Height (cm)	156.1 (5.6)	158.9 (5.4)	154.9 (5.3)	<0.001
Weight (kg)	57.3 (8.9)	58.2 (10.0)	56.9 (8.4)	<0.001
BMI (kg/m^2^)	23.5 (3.5)	23.0 (3.7)	23.7 (3.3)	<0.001
Hypertension (%)	1698 (17.7)	119 (4.3)	1579 (23.1)	<0.001
Diabetes mellitus (%)	625 (6.5)	43 (1.6)	582 (8.5)	<0.001
Hyperlipidemia (%)	617 (6.4)	41 (1.5)	576 (8.4)	<0.001
Kidney disease (%)	90 (0.9)	11 (0.4)	79 (1.2)	0.001
Smoking (%)	84 (0.9)	62 (2.2)	22 (0.3)	<0.001
Alcohol use (%)	456 (4.7)	273 (9.9)	183 (2.7)	<0.001

**Table 2:** Laboratory measurements of the patients (N = 9606).

Variable	All Patients (N = 9606)	Age Group		*p*
		≤50 Years (n = 2756)	>50 Years (n = 6850)	
SBP (mmHg)	124.8 (21.0)	116.1 (18.3)	128.3 (21.0)	<0.001
Albumin (g/dL)	4.35 (0.30)	4.37 (0.30)	4.34 (0.30)	<0.001
ALP (mg/dL)	76.3 (40.0)	64.5 (63.4)	81.1 (23.4)	<0.001
eGFR (mL/min/1.73 m^2^)	110.4 (24.5)	120.1 (23.7)	106.6 (23.8)	<0.001
Calcium (mg/dL)	2.24 (0.09)	2.21 (0.09)	2.25 (0.09)	<0.001
Glucose (mg/dL)	103.8 (21.5)	97.6 (16.6)	106.3 (22.8)	<0.001
GOT (mg/dL)	22.9 (13.1)	19.2 (9.4)	24.5 (14.0)	<0.001
HDL−C (mg/dL)	53.4 (14.7)	55.3 (14.9)	52.7 (14.5)	<0.001
LDL−C (mg/dL)	117.5 (31.4)	109.7 (29.0)	120.7 (31.7)	<0.001
Total cholesterol (mg/dL)	188.9 (36.1)	179.9 (33.0)	192.5 (36.6)	<0.001
Triglycerides (mg/dL)	104.4 (60.3)	88.5 (58.2)	110.9 (60.0)	<0.001
Hemoglobin (g/dL)	13.2 (1.3)	12.7 (1.5)	13.3 (1.1)	<0.001
Lumbar spine BMD (g/cm^2^)	0.883 (0.154)	0.994 (0.127)	0.838 (0.141)	<0.001
Right femoral neck BMD (g/cm^2^)	0.644 (0.115)	0.718 (0.111)	0.614 (0.103)	<0.001
Left femoral neck BMD (g/cm^2^)	0.653 (0.116)	0.727 (0.111)	0.624 (0.104)	<0.001

Values are mean (standard deviation) unless otherwise indicated. ALP: alkaline phosphatase; BMD: bone mineral density; BMI: body mass index; eGFR: estimated glomerular filtration rate; GOT: glutamic oxaloacetic transaminase; HDL-C: high-density lipoprotein cholesterol; LDL-C: low-density lipoprotein cholesterol; SBP: systolic blood pressure.

**Table 3:** Simple linear regression analysis of factors associated with bone mineral density of the lumbar spine in female patients with and without stratification by age.

Variable	Bone Mineral Density of the Lumbar Spine (g/cm^2^)					
	All (N = 9606)		Age ≤ 50 Years (n = 2756)		Age > 50 Years (n = 6850)	
	Std β	*p*	Std β	*p*	Std β	*p*
Hemoglobin (10 g/dL)	−0.084	<0.001	−0.072	<0.001	0.053	<0.001
Age (years)	−0.476	<0.001	−0.030	0.110	−0.283	<0.001
Height (cm)	0.322	<0.001	0.215	<0.001	0.207	<0.001
Weight (kg)	0.355	<0.001	0.395	<0.001	0.358	<0.001
BMI (kg/m^2^)	0.219	<0.001	0.328	<0.001	0.280	<0.001
Hypertension	−0.069	<0.001	0.052	0.006	0.036	0.003
Diabetes mellitus	0.001	0.883	0.073	<0.001	0.069	<0.001
Hyperlipidemia	−0.040	<0.001	0.057	0.003	0.016	0.187
Kidney disease	−0.021	0.043	0.008	0.685	−0.008	0.519
Smoking	0.061	<0.001	0.015	0.446	0.033	0.007
Alcohol use	0.116	<0.001	0.043	0.023	0.063	<0.001
SBP (10 mmHg)	−0.095	<0.001	0.113	<0.001	0.003	0.831
Albumin (10 g/dL)	0.005	0.641	−0.074	<0.001	0.004	0.753
ALP (10 mg/dL)	−0.193	<0.001	−0.129	<0.001	−0.154	<0.001
eGFR (10 mL/min/1.73 m^2^)	0.111	<0.001	−0.055	0.004	0.015	0.208
Calcium (10 mg/dL)	−0.105	<0.001	−0.101	<0.001	0.015	0.214
Glucose (10 mg/dL)	−0.014	0.180	0.083	<0.001	0.080	<0.001
GOT (10 mg/dL)	−0.087	<0.001	0.006	0.734	−0.005	0.656
HDL-C (10 mg/dL)	−0.029	0.004	−0.131	<0.001	−0.053	<0.001
LDL-C (10 mg/dL)	−0.060	<0.001	0.023	0.219	0.011	0.358
Total cholesterol (10 mg/dL)	−0.069	<0.001	−0.017	0.364	0.010	0.393
Triglycerides (10 mg/dL)	−0.005	0.642	0.077	<0.001	0.084	<0.001

ALP: alkaline phosphatase; BMI: body mass index; eGFR: estimated glomerular filtration rate; GOT: glutamic oxaloacetic transaminase; HDL-C: high-density lipoprotein cholesterol; LDL-C: low-density lipoprotein cholesterol; SBP: systolic blood pressure.

**Table 4:** Simple linear regression analysis of factors associated with bone mineral density of the right femoral neck in all female patients and those stratified by age.

Variable	Bone Mineral Density of the Right Femoral Neck (g/cm^2^)					
	All (N = 9606)		Age ≤ 50 Years (n = 2756)		Age > 50 Years (n = 6850)	
	Std β	*p*	Std β	*p*	Std β	*p*
Hemoglobin (10 g/dL)	−0.061	<0.001	−0.065	0.001	0.080	<0.001
Age (years)	−0.461	<0.001	−0.075	<0.001	−0.325	<0.001
Height (cm)	0.357	<0.001	0.234	<0.001	0.273	<0.001
Weight (kg)	0.391	<0.001	0.423	<0.001	0.389	<0.001
BMI (kg/m^2^)	0.239	<0.001	0.349	<0.001	0.279	<0.001
Hypertension	−0.085	<0.001	0.055	0.004	−0.003	0.819
Diabetes mellitus	−0.001	0.942	0.096	<0.001	0.053	<0.001
Hyperlipidemia	−0.049	<0.001	0.033	0.079	−0.002	0.853
Kidney disease	−0.016	0.117	0.013	0.504	−0.005	0.651
Smoking	0.053	<0.001	0.003	0.880	0.034	0.005
Alcohol use	0.115	<0.001	0.055	0.004	0.063	<0.001
SBP (10 mmHg)	−0.096	<0.001	0.119	<0.001	−0.027	0.025
Albumin (10 g/dL)	−0.004	0.723	−0.084	<0.001	0.004	0.763
ALP (10 mg/dL)	−0.171	<0.001	−0.130	<0.001	−0.103	<0.001
eGFR (10 mL/min/1.73 m^2^)	0.131	<0.001	−0.010	0.605	0.052	<0.001
Calcium (10 mg/dL)	−0.091	<0.001	−0.083	<0.001	0.021	0.077
Glucose (10 mg/dL)	−0.008	0.412	0.087	<0.001	0.072	<0.001
GOT (10 mg/dL)	−0.062	<0.001	0.025	0.196	0.012	0.324
HDL-C (10 mg/dL)	−0.049	<0.001	-0.139	<0.001	−0.068	<0.001
LDL-C (10 mg/dL)	−0.033	0.001	0.014	0.476	0.045	<0.001
Total cholesterol (10 mg/dL)	−0.056	<0.001	-0.034	0.074	0.027	0.024
Triglycerides (10 mg/dL)	0.001	0.887	0.086	<0.001	0.075	<0.001

ALP: alkaline phosphatase; BMI: body mass index; eGFR: estimated glomerular filtration rate; GOT: glutamic oxaloacetic transaminase; HDL-C: high-density lipoprotein cholesterol; LDL-C: low-density lipoprotein cholesterol; SBP: systolic blood pressure.

**Table 5:** Simple linear regression analysis of factors associated with bone mineral density of the left femoral neck in all female patients and those stratified by age.

Variable	Bone Mineral Density of the Left Femoral Neck (g/cm^2^)					
	All (N = 9606)		Age ≤ 50 years (n = 2756)		Age > 50 years (n = 6850)	
	Std β	*P*	Std β	*P*	Std β	*P*
Hemoglobin (10 g/dL)	−0.063	<0.001	−0.061	0.001	0.072	<0.001
Age (years)	−0.455	<0.001	−0.053	0.005	−0.328	<0.001
Height (cm)	0.363	<0.001	0.251	<0.001	0.277	<0.001
Weight (kg)	0.402	<0.001	0.444	<0.001	0.395	<0.001
BMI (kg/m^2^)	0.247	<0.001	0.364	<0.001	0.283	<0.001
Hypertension	−0.086	<0.001	0.045	0.019	−0.004	0.772
Diabetes mellitus	−0.001	0.957	0.077	<0.001	0.055	<0.001
Hyperlipidemia	−0.042	<0.001	0.035	0.067	0.006	0.596
Kidney disease	−0.014	0.159	0.018	0.343	−0.005	0.697
Smoking	0.054	<0.001	0.008	0.673	0.034	0.005
Alcohol use	0.115	<0.001	0.048	0.011	0.071	<0.001
SBP (10 mmHg)	−0.097	<0.001	0.117	<0.001	−0.030	0.014
Albumin (10 g/dL)	0.004	0.682	−0.078	<0.001	0.013	0.286
ALP (10 mg/dL)	−0.175	<0.001	−0.127	<0.001	−0.118	<0.001
eGFR (10 mL/min/1.73 m^2^)	0.128	<0.001	−0.017	0.364	0.052	<0.001
Calcium (10 mg/dL)	−0.083	<0.001	−0.078	<0.001	0.030	0.012
Glucose (10 mg/dL)	−0.007	0.468	0.076	<0.001	0.075	<0.001
GOT (10 mg/dL)	−0.067	<0.001	0.028	0.145	0.003	0.827
HDL-C (10 mg/dL)	−0.042	<0.001	−0.138	<0.001	−0.161	<0.001
LDL-C (10 mg/dL)	−0.028	0.006	0.036	0.057	0.042	0.001
Total cholesterol (10 mg/dL)	−0.050	<0.001	−0.012	0.523	0.027	0.026
Triglycerides (10 mg/dL)	−0.007	0.477	0.087	<0.001	0.059	<0.001

ALP: alkaline phosphatase; BMI: body mass index; eGFR: estimated glomerular filtration rate; GOT: glutamic oxaloacetic transaminase; DL-C: high-density lipoprotein cholesterol; LDL-C: low-density lipoprotein cholesterol; SBP: systolic blood pressure.

**Table 6:** Multiple linear regression analysis of factors associated with bone mineral density of the lumbar spine in female patients with and without stratification by age group.

Variable	Bone Mineral Density of the Lumbar Spine (g/cm^2^)					
	All (N = 9606)		Age ≤ 50 Years (n = 2756)		Age > 50 Years (n = 6850)	
	Std β	*p*	Std β	*p*	Std β	*p*
Hemoglobin (10 g/dL)	−0.025	0.005	−0.077	<0.001	0.046	<0.001
Age (years)	−0.511	<0.001	−0.077	<0.001	−0.333	<0.001
BMI (kg/m^2^)	0.270	<0.001	0.324	<0.001	0.281	<0.001
Alcohol use	0.018	0.033	0.037	0.042	0.027	0.014
SBP (10 mmHg)	0.020	0.034			0.031	0.006
Albumin (10 g/dL)	−0.050	<0.001			−0.084	<0.001
ALP (10 mg/dL)	−0.142	<0.001	−0.140	<0.001	−0.207	<0.001
eGFR (10 mL/min/1.73 m^2^)	−0.018	0.048	−0.043	0.019	−0.026	0.023
Calcium (10 mg/dL)			−0.056	0.003	0.027	0.022
Glucose (10 mg/dL)	0.055	<0.001	0.071	<0.001	0.057	<0.001
HDL-C (10 mg/dL)	0.026	0.008			0.031	0.031
LDL-C (10 mg/dL)	−0.030	0.001			−0.031	0.005
Triglycerides (10 mg/dL)	0.036	<0.001			0.042	0.042
Model adjusted *R^2^*	0.330		0.148		0.214	

ALP: alkaline phosphatase; BMI: body mass index; eGFR: estimated glomerular filtration rate; Hb: hemoglobin; HDL-C: high-density lipoprotein cholesterol; LDL-C: low-density lipoprotein cholesterol; SBP: systolic blood pressure. Variables listed in the table include only those retained in the final models. Other variables evaluated during model development included smoking, glutamic oxaloacetic transaminase, and total cholesterol.

**Table 7:** Multiple linear regression analysis of factors associated with bone mineral density of the right femoral neck in female patients with and without stratification by age group.

Variable	Bone Mineral Density of the Right Femoral Neck (g/cm^2^)					
	All (N = 9606)		Age ≤ 50 Years (n = 2756)		Age > 50 Years (n = 6850)	
	Std β	*p*	Std β	*p*	Std β	*p*
Hemoglobin (10 g/dL)			−0.065	<0.001	0.056	<0.001
Age (years)	−0.491	<0.001	−0.117	<0.001	−0.360	<0.001
BMI (kg/m^2^)	0.288	<0.001	0.360	<0.001	0.286	<0.001
Alcohol use	0.020	0.021	0.043	0.015	0.024	0.024
Albumin (10 g/dL)	−0.056	<0.001	−0.053	0.004	−0.077	<0.001
ALP (10 mg/dL)	−0.124	<0.001	−0.158	<0.001	−0.157	<0.001
Calcium (10 mg/dL)					0.026	0.023
Glucose (10 mg/dL)	0.053	<0.001	0.076	<0.001	0.051	<0.001
GOT (10 mg/dL)					0.029	0.009
LDL-C (10 mg/dL)			−0.043	0.021		
Model adjusted *R^2^*	0.319		0.172		0.224	

ALP: alkaline phosphatase; BMI: body mass index; GOT: glutamic oxaloacetic transaminase; LDL-C: low-density lipoprotein cholesterol. Variables listed in the table include only those retained in the final models. Other variables evaluated during model development and found to be not significant included estimated glomerular filtration rate, high-density lipoprotein cholesterol, smoking, systolic blood pressure, total cholesterol, and triglycerides.

**Table 8:** Multiple linear regression analysis of factors associated with bone mineral density of the left femoral neck in female patients with and without stratification by age group.

Variable	Bone Mineral Density of the Left Femoral Neck (g/cm^2^)					
	All (N = 9606)		Age ≤ 50 Years (n = 2756)		Age > 50 Years (n = 6850)	
	Std β	*p*	Std β	*p*	Std β	*p*
Hemoglobin (10 g/dL)			−0.070	<0.001	0.050	<0.001
Age (years)	−0.484	<0.001	−0.100	<0.001	−0.361	<0.001
BMI (kg/m^2^)	0.297	<0.001	0.369	<0.001	0.292	<0.001
Alcohol use	0.022	0.012	0.040	0.025	0.031	0.004
Albumin (10 g/dL)	−0.047	<0.001	−0.043	0.017	−0.073	<0.001
ALP (10 mg/dL)	−0.128	<0.001	−0.150	<0.001	−0.170	<0.001
Calcium (10 mg/dL)					0.037	0.001
Glucose (10 mg/dL)	0.050	<0.001	0.052	0.005	0.057	<0.001
Model adjusted *R^2^*	0.318		0.172		0.231	

ALP: alkaline phosphatase; BMI: body mass index. Variables listed in the table include only those retained in the final models. Other variables evaluated during model development and found not significant included estimated glomerular filtration rate, glutamic oxaloacetic transaminase, high-density lipoprotein cholesterol, low-density lipoprotein cholesterol, smoking, systolic blood pressure, total cholesterol, and triglycerides.

## Data Availability

The data used to support the findings of this study are available from the corresponding author upon request.
